# Applicability study of AI attribution methods for ophthalmic image classification

**DOI:** 10.1038/s41598-025-33120-5

**Published:** 2026-01-05

**Authors:** Ali Yavari, Tilman Schmoll, Rainer A. Leitgeb, Kim Lien Huber, Heiko Stino, Andreas Pollreisz, Wolfgang Drexler, Thomas Schlegl

**Affiliations:** 1 Center for Medical Physics and Biomedical Engineering, Medical University of Vienna, Waehringer Guertel 18-20 (4L), 1090 Vienna, Austria; 2 Carl Zeiss Meditec AG, Oberkochen, Germany; 3 Department of Ophthalmology and Optometry, Medical University of Vienna, Waehringer Guertel 18-20, 1090 Vienna, Austria

**Keywords:** Computational biology and bioinformatics, Diseases, Health care, Mathematics and computing, Medical research

## Abstract

Optical coherence tomography (OCT) enables early detection of vision-threatening diabetic retinopathy (DR) and retinal fluid accumulation, both major complications of diabetes. Despite high classification performance, deep learning models face limited clinical adoption due to poor interpretability. While attribution methods are effective for explaining predictions in the natural image domain, their applicability to medical imaging remains underexplored. To bridge this gap, our work explores how well these strong results transfer to the medical imaging domain. This study evaluates three cutting-edge attribution methods— DeepLIFT, AGI, and AttEXplore —for explaining predictions of a VGG16-based deep learning model in DR classification using widefield OCTA en face images and fluid detection in OCT B-scans. We assess attribution methods’ ability to highlight clinically relevant regions using quantitative (insertion and deletion scores) and qualitative (heatmap visual analysis) measures. Although the VGG16 model achieves high classification accuracy (94% for DR and 98% for fluid), attribution methods yield markedly different qualitative results due to variations in underlying assumptions and hyperparameter sensitivity. Additionally, high insertion or low deletion scores do not necessarily correlate with clinically meaningful visual attributions. In particular, insertion- and deletion-based behaviour can be more informative in pathological cases, where localized lesions can drive predictions, but tends to be less informative in normal cases, where confirming the absence of pathology requires global contextual evidence. Comparing the three approaches, we find that AGI and AttEXplore achieve similarly strong quantitative performance, whereas AttEXplore more consistently highlights clinically meaningful structures in pathological cases than AGI and DeepLIFT, making it the preferred option in our DR and fluid detection settings. However, our results also show that in any potential clinical usage, all three methods must be interpreted alongside clinical expertise, and even the result of the best-performing attribution approach cannot serve as an automatic proxy for clinical relevance. This study provides critical insights into challenges of applying attribution methods to medical imaging, using ophthalmic data, laying a foundation for improving transparency and trust in AI-assisted diagnostics.

## Introduction

Deep neural networks (DNNs) have rapidly become crucial in many machine learning applications, significantly enhancing predictive accuracy across diverse tasks. Despite these achievements, the inherently complex and nonlinear structure of DNNs makes their decision-making processes opaque, prompting the critical need for effective attribution methods to interpret model predictions. Attribution methods play a fundamental role in elucidating how input features contribute to model outputs, fostering trustworthiness and transparency required for broader adoption in sensitive domains^1^. However, attribution methods, ranging from traditional techniques to more advanced formulations, share fundamental limitations. These limitations arise from their dependence on assumptions that may not align with the complex behavior of deep neural networks or hold consistently across contexts. Whether through manual reference choices, fixed integration paths, or rigid treatment of feature interactions, these methods risk introducing bias and reducing interpretive reliability^2^. Regardless of their varying levels of sophistication, they commonly struggle to fully capture the complex, nonlinear decision dynamics of deep neural networks^3^.

These challenges become even more critical in high-stakes fields like medical imaging, where interpretability directly impacts human health. In the medical imaging domain, attribution methods face significant challenges and strict clinical requirements. Firstly, explanations must be trustworthy and clinically relevant, misleading or incorrect attributions, such as highlighting irrelevant areas in ophthalmic images, can endanger patient safety and erode clinician trust. Secondly, medical professionals require explanations that align with clinical knowledge and workflows, which often demands domain-specific visualization and annotation of anatomical features to ensure interpretability. Lastly, the high stakes of medical decisions necessitate transparent Artificial Intelligence (AI) systems that can be audited and regulated, particularly to prevent reliance on spurious correlations or biases in the data^4^.

The primary motivation of our work is to assess the gap between high-performance deep learning models and their clinical applicability in ophthalmology by examining a fundamental challenge: the interpretability of AI-based diagnostic models. We attempt to answer the following research questions: (1) Can the outputs of attribution methods be trusted in medical imaging tasks? (2) Do visual explanation maps provided by Attribution Methods align with the clinically most relevant imaging features and image regions? (3) Do standard quantitative metrics, such as insertion or deletion scores, reliably reflect the quality of qualitative visual explanations? (4) Do different Attribution Methods yield the same visual explanation maps for the same underlying discriminative model? (5) Are these methods truly ready-to-use tools, or do they require careful hyperparameter tuning to yield meaningful results?

In this study, we evaluate three attribution methods: Deep Learning Important FeaTures (DeepLIFT)^5^, using baseline differences for feature importance; Adversarial Gradient Integration (AGI)^6^, integrating gradients along adversarial paths for reference-free explanations; and Attribution method for Explanation with model parameter eXploration (AttEXplore)^3^, combining frequency-domain perturbations and model exploration for high-resolution maps. These methods were selected to span traditional to cutting-edge approaches, ensuring a comprehensive analysis in medical imaging. To address our research questions, we propose a dual-pronged workflow that integrates quantitative evaluations (insertion and deletion scores) and qualitative evaluations (visual analysis). We apply the attribution methods to diabetic retinopathy (DR) classification and retinal fluid detection using a VGG16 backbone^7^. The data source is optical coherence tomography (OCT) B-scans and widefield OCT angiography (OCTA) en face images, which are of high diagnostic relevance in the case of DR. Via the workflow, we analysis generated heatmaps visually to check if clinically relevant regions are accurately highlighted (RQ1 and RQ2). We calculate the insertion and deletion scores, state-of-the-art quantitative measures for attribution methods, for selected samples, and then check whether these scores align with any clinically relevant regions (RQ3). Each attribution method is leveraged to explain the same VGG16-based classifier (RQ4). We also design some experiments to investigate the sensitivity of the studied attribution methods to their hyperparameters (RQ5).

Among state-of-the-art attribution methods, local surrogate models like Local Interpretable Model-agnostic Explanations (LIME)^8^ pioneered interpretable attributions by fitting simple, transparent models (e.g., linear regressions or decision trees) around individual instances to approximate complex model behavior locally. DeepLIFT^5^ extended this paradigm by propagating activation differences relative to reference inputs backward through a network, thereby assigning each feature a contribution score that reflects its impact on the final prediction. Gradient-based approaches, notably Integrated Gradients (IG)^9^, introduced an axiomatic framework to guarantee that attributions satisfy desirable properties, such as sensitivity and implementation invariance, by integrating gradients along paths from a baseline input to the target instance. More recent advancements have explored hybrid and boundary-aware strategies: AGI^6^ enhances attribution by integrating gradients along adversarially guided paths, eliminating the need for a reference input and focusing on class discrimination. AttEXplore^3^ augments gradient-based explanations with adversarial perturbations and systematic parameter exploration to improve attribution precision. In contrast, More Faithful and Accelerated Boundary-based Attribution (MFABA)^10^ accelerates faithful boundary-based attributions by tracking how classification decisions change near decision boundaries. DeepLIFT, a well-known method, has been widely applied in medical imaging to provide feature importance, whereas AGI and AttEXplore have demonstrated, in experiments conducted on natural image domains, the ability to generate more precise and robust attributions^3,6^. Given that both approaches systematically explore model decision boundaries, they may offer even greater potential for impactful application in the domain of medical image analysis.

Several traditional attribution methods, including DeepLIFT, have been applied by researchers in medical imaging: Lopatina et al. used DeepLIFT attribution combined with perturbation analysis to interpret a convolutional neural network (CNN) trained on susceptibility-weighted Magnetic Resonance (MR) images for multiple sclerosis classification. Their analysis revealed relevance patterns centered on venous structures and specific brain regions^11^. Souza Jr. et al.^12^ employed five attribution methods—saliency, input $\times$ gradients^13^, guided backpropagation^14^, IG, and DeepLIFT—to interpret CNN-based classification of early esophageal cancer in Barrett’s esophagus. Their results showed that saliency maps aligned best with expert annotations. Eitel et al. evaluated guided backpropagation and Layer-wise Relevance Propagation (LRP)^15^ in Magnetic Resonance Imaging (MRI)-based Alzheimer’s classification, revealing robustness challenges^16^. Nishizawa et al.^17^ used LRP and IG for MRI-based temporomandibular joint disorder heatmaps, while Ma et al.^18^ applied LRP for caries prediction. Li et al.^19^ demonstrated clinical utility by integrating Shapley Additive exPlanations (SHAP)^20^ values with decision curve analysis for renal cell carcinoma outcomes from Computed Tomography (CT) images. These studies illustrate the utility of established attribution methods in medical imaging, laying groundwork for emerging techniques like AGI and AttEXplore, which could enhance interpretability in future work.

To ground our investigation in clinically significant conditions, we focus on DR, which involves complex microvascular alterations, and diabetic macular edema (DME), marked by fluid accumulation in the retina. DR is the primary microvascular complication among working-age adults with diabetes mellitus (DM), posing a major threat to vision^21^ and expected to affect 191 million people globally by 2030^22,23^. Approximately 35% of diabetic individuals develop some form of DR, with 7% progressing to advanced stages, spanning from nonproliferative to proliferative forms^24,25^. DME, a vision-threatening manifestation of DR, involves the accumulation of fluid in the central retina and affects around 750,000 people over 40 in the U.S. alone^26^. This fluid buildup, detectable via OCT, correlates strongly with visual impairment and guides treatment using anti-Vascular Endothelial Growth Factor (VEGF) therapy, where fluid resolution often leads to improved vision. Given the often silent progression of these conditions, regular ophthalmologic screening, including attention to peripheral lesions and wide field of view (FoV) imaging, is essential for early detection and effective disease monitoring^27–29^. OCTA, an extension of OCT^30^, offers non-invasive visualization of retinal microvasculature blood flow^31,32^. Widefield OCTA, particularly in modern Swept-Source (SS)-OCT systems, delivers significant clinical advantages^33–35^.

In terms of practical DR and fluid detection, state-of-the-art classification models have demonstrated promising performance in evaluation studies, though further clinical validation is required before such models can be adopted in routine practice. A fully automated CNN-based algorithm on OCTA images for detecting DR was presented in^36^, reporting high accuracy, sensitivity, and specificity. Zang et al.^37^ offered deep learning frameworks for the automated detection of DR, age-related macular degeneration (AMD), and glaucoma based on structural and angiographic OCT data volumes. Kermany^26^ presents a deep learning framework capable of accurately identifying and differentiating intraretinal and subretinal fluid in OCT images, enabling reliable detection of fluid-associated retinal abnormalities.

As the main contributions of this work: We (i) perform a systematic comparison of three representative attribution methods, DeepLIFT, AGI, and AttEXplore, within a unified VGG16 framework for DR classification and fluid detection tasks, (ii) pair complementary quantitative metrics with visually analyzed heatmaps to evaluate each method’s ability to localize clinically relevant retinal abnormalities and to assess the agreement between the two metrics, (iii) analyze the sensitivity of the attribution methods to their specific hyperparameters to examine their applicability in clinical settings. More broadly, our study provides a focused evaluation of attribution methods in retinal imaging and highlights key considerations for applying Explainable AI (XAI) techniques in clinical contexts.

## Data

In this section, we describe the two ophthalmic imaging datasets, widefield OCTA en face images for DR classification and OCT B-scans for retinal fluid detection, along with the data notation used in our analysis. None of the datasets contain pixel-level annotations; in particular, OCT B-scans lack pixel-wise fluid labels, and OCTA en face images do not include lesion-type annotations.

**Fig. 1:**
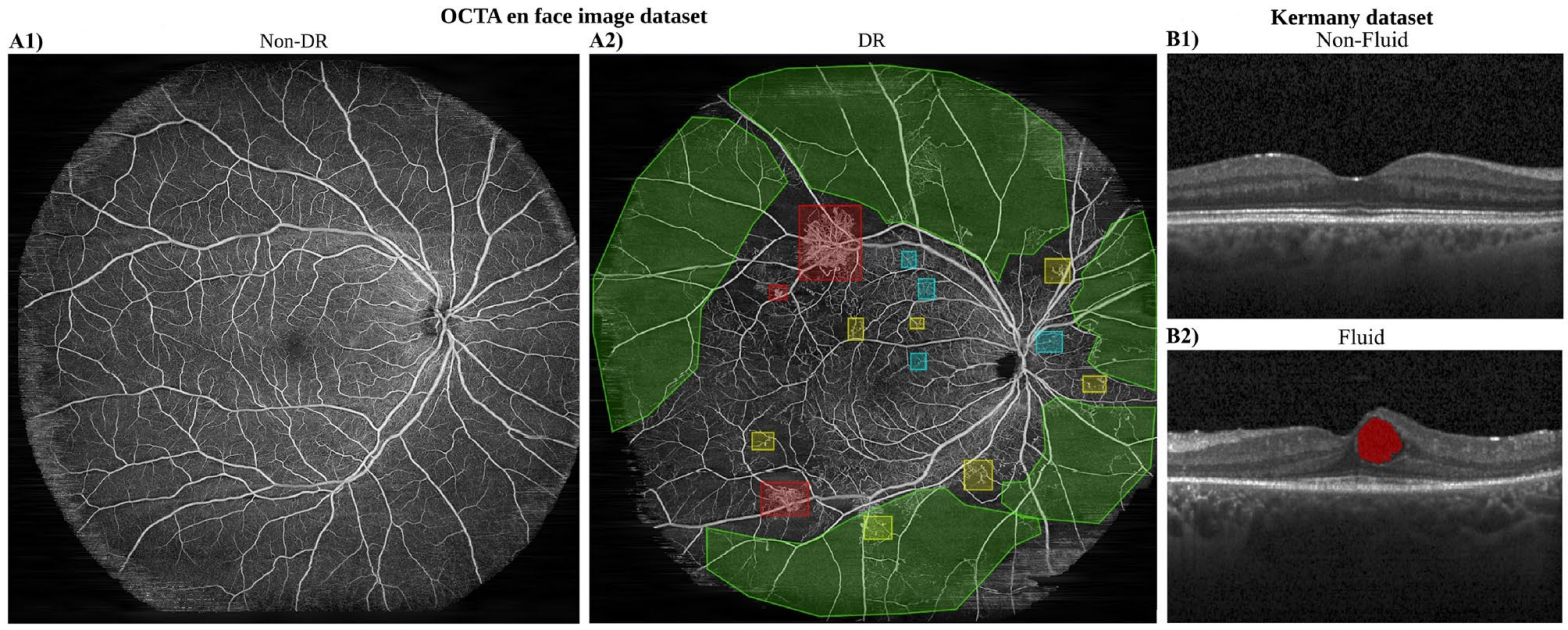
Widefield OCTA en face images and OCT B-scans illustrating different retinal conditions. (**A1**) Non-DR case with an intact vascular structure. (**A2**) DR case with exemplary annotations: neovascularizations (NV, red), microaneurysms (MA, cyan), intraretinal microvascular abnormalities (IRMA, yellow), and non-perfusion areas (NPA, green). (**B1**) Non-fluid B-scan. (**B2**) Fluid B-scan with retinal thickening and fluid accumulation (fluid pocket labeled in red).

### Widefield OCTA en face image dataset and data collection.

OCTA is a non-invasive imaging modality that visualizes retinal microvasculature by detecting motion contrast of erythrocytes through repeated B-scans acquisitions at the same lateral location. In this dataset, OCTA contrast was derived using complex variance analysis from pairs of repeated B-scans acquired at each location. The volumetric data were processed to generate en face projections using maximum intensity projection (MIP) from a slab extending from the inner limiting membrane (ILM) to 20 µm above the retinal pigment epithelium (RPE). This approach effectively captures both the superficial and deep capillary plexuses. The resulting en face OCTA images provide high-contrast, two-dimensional views of the retinal vasculature across the macula and mid-peripheral retina. These images enable assessment of pathologies such as capillary dropout, neovascularization, and microaneurysms^38^. The OCTA dataset was acquired using a modified PLEX^®^ Elite 9000 swept-source OCT system^34^ equipped with an FDML laser operating at an axial scan rate of 1.68 MHz. The axial resolution was 9 µm FWHM in tissue, and the lateral resolution was 20 µm (defined at the 1/e$^{2}$ beam diameter). Both eyes of each participant were scanned with two repeated B-scans per lateral position. The acquired volumes consisted of 1536 $\times$ 2048 $\times$ 2044 samples, corresponding to approximately 6 mm $\times$ 18 mm $\times$ 18 mm (zxy) on the retina. Healthy volunteers were also included in the clinical study, which was approved by the local ethics committee of the Medical University of Vienna. Participants provided informed consent for data use following a physician-led briefing. The study was conducted in accordance with relevant guidelines and regulations and adhered to the principles of the declaration of Helsinki. Widefield OCTA scans were acquired by trained personnel following a standardized protocol. The dataset included 348 en face OCTA images from 90 diabetic patients and 37 healthy volunteers, comprising 252 DR and 96 non-DR images. Both eyes were scanned for each patient, and in some cases, multiple images per eye were acquired. Some patients have follow-up scans, but not all; multiple images per eye may originate from the same session or from different visits. All images from a given patient were exclusively assigned to either the training or the validation set, with no overlap between the two. Fig. 1A1 shows a non-DR retina with intact vasculature, while Fig. 1A2 displays a DR case exhibiting neovascularization (NV), non-perfusion areas (NPA), intraretinal microvascular abnormalities (IRMA), and microaneurysms (MA).

### Kermany OCT B-scan dataset

The Kermany OCT dataset originally comprises retinal B-scans from multiple sources with varying fields of view^26^. To ensure consistency, we standardized the dataset by retaining only B-scans with a uniform 6 $\times$ 6mm field of view. Additionally, we curated the dataset by excluding all B-scans of OCT scans that showed signs of Choroidal Neovascularization (CNV) or Drusen. B-scan labels focus solely on the presence or absence of fluid. In our experiments, a B-scan is labeled as non-fluid only if it contains no signs of fluid. Importantly, curated B-scans (fluid and non-fluid) may still exhibit other disease manifestations. The final dataset includes 2,137 retinal B-scans, categorized into non-fluid (1,387 B-scans from 329 patients) and fluid (750 B-scans from 172 patients) B-scans, effectively making this a binary classification task of fluid versus non-fluid B-scans. The dataset was systematically divided into three subsets: Training (1,370 B-scans), Test (440 B-scans), and Validation (327 B-scans). To avoid overlap, B-scans from the same patient were included in only one dataset, either training or validation. Fig. 1B1 illustrates a non-fluid retina with an intact structure, while Fig. 1B2 presents a fluid B-scan characterized by retinal fluid, labeled as red.

### Data notation

Each sample in our study is represented as a pair $\bigl (\textbf{X}^{(i)}, y^{(i)}\bigr )$, where $i \in \{1, \dots , T\}$ indexes the sample and $T$ is the total number of 2D images. Specifically, for the widefield OCTA dataset, each en face image $\textbf{X}_{\textrm{OCTA}}^{(i)} \in \mathbb {R}^{H_O \times W_O}$, where $H_O$ and $W_O$ denote the image height and width in pixels, respectively, corresponds to a single volumetric scan. In contrast, for the Kermany dataset, each B-scan $\textbf{X}_{\textrm{K}}^{(i)} \in \mathbb {R}^{H_K \times W_K}$, with $H_K$ and $W_K$ its own height and width, is one slice extracted from a volumetric OCT volume. Thus the full dataset comprises $T = \sum _{j=1}^{P} M^{(j)}$ B-scans from $P$ patient volumes, where $M^{(j)}$ denotes the number of B-scans in the $j$-th patient volume. Each image $\textbf{X}^{(i)}$ is paired with a label $y^{(i)} \in \{0,1\}$ indicating the absence ($0$) or presence ($1$) of the pathology of interest (DR for OCTA, fluid for Kermany), providing the basis for our binary classification tasks.

## Methods

**Fig. 2:**
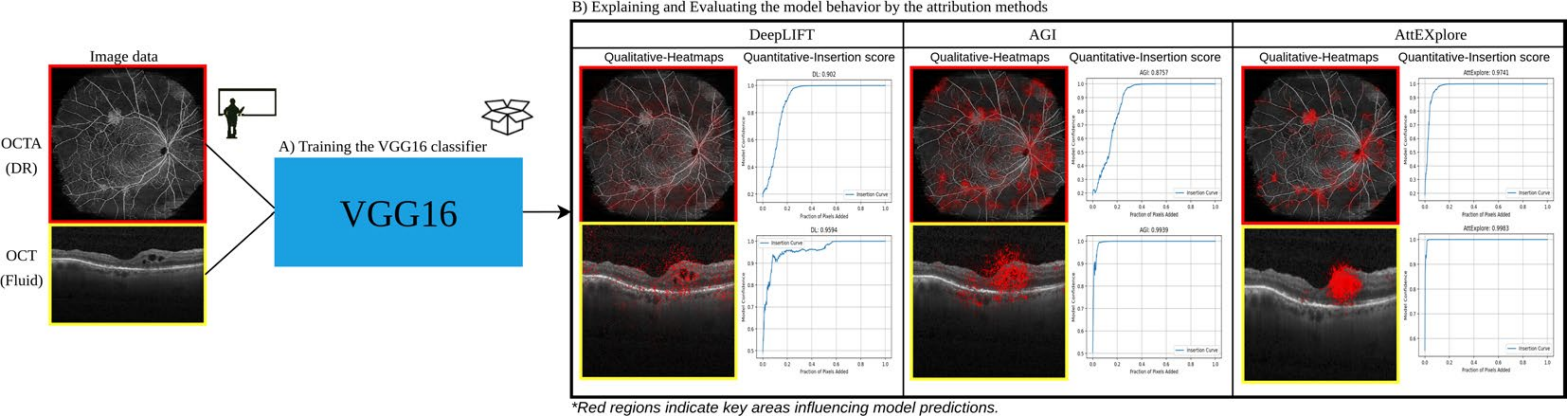
Depending on the experiment, OCT or OCTA images are processed by a VGG16-based classifier for DR classification and fluid detection (**A**). Model interpretability is then analyzed using three attribution methods: DeepLIFT (Deep Learning Important FeaTures), AGI (Adversarial Gradient Integration), and AttEXplore (Attribution method for Explanation with model parameter eXploration) (**B**).

In this section, we apply three attribution techniques, DeepLIFT, AGI, and AttEXplore, to ophthalmic image classification tasks, where precise localization of clinically relevant features is critical. Each method is incorporated into a VGG16-based binary classification pipeline and assessed for its capability to produce interpretable heatmaps on two datasets: widefield OCTA en face images and Kermany OCT B-scans. Fig.  2 outlines the overall methodology. The following subsections first describe the VGG16-based classification pipeline, then detail each attribution method. Finally, we explain the quantitative (insertion and deletion scores) and qualitative (visual analysis) metrics used to evaluate attribution performance.

### Image-level binary classification model.

For the binary medical image classification task, we use all convolutional layers of a VGG16 model pre-trained on ImageNet. The network relies on the VGG16 convolutional backbone, which consists of five convolutional blocks. Blocks 1 and 2 contain two convolutional layers each, while blocks 3 to 5 contain three layers. All layers use ReLU activation, and each block is followed by a max-pooling layer to progressively reduce spatial dimensions and extract hierarchical features. The classification head includes a fully connected layer with 256 units and ReLU activation, a dropout layer with a rate of 0.5 to mitigate overfitting, and a final output layer with a single neuron and sigmoid activation to generate the probability of the positive class. In our study, we selected the VGG16 architecture because it achieves high accuracy while also being time-efficient, which is essential in a clinical setting where both accuracy and speed are key metrics. Importantly, this approach avoids unnecessary model complexity, making it well-suited for practical deployment. All attribution methods are applied consistently to the same base model, ensuring a fair comparison.

*Training the Network.* During training, we fine-tune the model weights on the specific ophthalmology datasets $(\textbf{X}, y)$, where each sample is $(\textbf{X}^{(i)}, y^{(i)})$. We apply the Adam optimizer^39^ and use focal binary cross-entropy loss (FL)^40^ defined in Eq.  1, where $y^{(i)} \in \{0,1\}$ is the ground-truth label, $\hat{p}^{(i)}$ is the predicted probability of $y^{(i)}=1$, and $p_t^{(i)} = \hat{p}^{(i)}$ if $y^{(i)}=1$ otherwise $1-\hat{p}^{(i)}$. We fix $\gamma = 2$ as recommended in^40^ and compute $\alpha$ from the training data as the ratio of the minority class frequency to the total frequency (e.g., if class 1 is less frequent than class 0, $\alpha = \frac{\text {weight}_{\text {class 1}}}{\text {weight}_{\text {class 0}} + \text {weight}_{\text {class 1}}}$). All predictions $\hat{p}^{(i)}$ arise from applying a sigmoid activation to the model’s final output, thereby providing a probability estimate for each image $X^{(i)}.$

1 $$\begin{aligned} FL\bigl (p_t^{(i)}\bigr ) = -\alpha \bigl (1 - p_t^{(i)}\bigr )^\gamma \log \bigl (p_t^{(i)}\bigr ) - (1 - \alpha ) \bigl (p_t^{(i)}\bigr )^\gamma \log \bigl (1 - p_t^{(i)}\bigr ) \end{aligned}$$

### Explaining and evaluating model decisions using attribution methods

*Attribution of Predictions in ML Models.* Formally, consider an input instance $\textbf{X}^{(i)} = \bigl (x^{(i)}_1, \dots , x^{(i)}_n\bigr ) \in \mathbb {R}^n$, where $n = H \cdot W$ is the total number of pixels in a 2D image of height $H$ and width $W$. This formulation assumes that the original image has been flattened into a 1D vector. Attribution in machine learning aims to quantify how each input feature influences a model’s output. Let $f: \mathbb {R}^n \rightarrow \mathbb {R}$ be a function that returns a real-valued score (e.g., a logit or predicted probability) for a given input $\textbf{X}^{(i)}$. The attribution $\textbf{A}_f\bigl (\textbf{X}^{(i)}\bigr )$ is defined as a vector $(a_1, \dots , a_n) \in \mathbb {R}^n$, where each component $a_j$ represents the contribution of input feature $x^{(i)}_j$ to the output $f(\textbf{X}^{(i)})$. For methods that require a reference input—such as DeepLIFT—attribution is instead computed relative to a baseline $\mathbf {X'}^{(i)} \in \mathbb {R}^n$ of the same dimensionality, i.e., $\textbf{A}_f\bigl (\textbf{X}^{(i)}, \mathbf {X'}^{(i)}\bigr )$. These attributions help identify which input dimensions most influence the model’s decision^9^. While $A$ may appear as a scalar in the method-specific formulations that follow, it reflects a single feature’s effect; computing all $a_j$ values yields the full attribution vector, which can be reshaped into a 2D map for visualization.

*DeepLIFT.* DeepLIFT assigns importance scores to input pixels by comparing the network’s response to a designated reference input. Instead of relying solely on local gradients, DeepLIFT propagates *differences-from-reference* activation values backward through the network using a set of rules that preserve the “summation-to-delta” property^5^. The contributions calculated by DeepLIFT satisfy the condition shown in Eq. 2, where $f(\textbf{X}'^{(i)})$ is the model’s output at the baseline input $\mathbf {X'}^{(i)}$. By summing over finite differences rather than relying on infinitesimal gradients, DeepLIFT highlights relevant pixels even in regions where gradients vanish or saturate. The underlying motivation for this approach is that input pixels may still meaningfully contribute to the output, even when the gradients of intermediate activations are zero—for example, due to saturation. In such cases, gradients alone fail to capture the input’s true influence, whereas DeepLIFT explicitly measures how the activation changes relative to the reference input, thereby enabling continuous and interpretable attribution.

2 $$\begin{aligned} A = \sum _{p=1}^n a_p = f\bigl (\textbf{X}^{(i)}\bigr ) - f\bigl (\mathbf {X'}^{(i)}\bigr ) \quad \text {([5])} \end{aligned}$$

*AGI.* AGI is a targeted attribution method that computes pixel contributions by integrating gradients along adversarial trajectories, without requiring a predefined reference input. Given a sample $(\textbf{X}^{(i)}, y^{(i)})$, AGI selects a specific false class $c \ne y^{(i)}$ and constructs a targeted adversarial path by iteratively updating the input $\textbf{X}^{(i)}$ to increase the model’s output $f^c(\textbf{X}^{(i)})$ for the target class $c$. At each iteration $j \in \{1, \dots , m_{iter}\}$, where $m_{iter}$ is the maximum number of adversarial steps, the input is updated along the normalized gradient direction of $f^c$, scaled by a step size $\eta$. Simultaneously, attribution is accumulated using the gradient of the true class output $f^{y^{(i)}}(\textbf{X}^{(i)}_j)$, evaluated at the current adversarial input $\textbf{X}^{(i)}_j$. As shown in Eq. 3, this results in a cumulative attribution $A$ that reflects how input pixels contribute to discriminating the true class from the target false class. The $\text {sign}(\cdot )$ term extracts the unit ascent direction, and the denominator normalizes the gradient magnitude. Unlike IG, AGI follows a curved path determined by the steepest ascent toward a target class, enabling input-specific, reference-free explanations that trace decision boundaries in feature space.

3 $$\begin{aligned}&A = - \sum _{j=1}^{m_{iter}} \nabla _{\textbf{X}^{(i)}_j} f^{y^{(i)}}\bigl (\textbf{X}^{(i)}_j\bigr ) \cdot \Bigl ( \eta \cdot \text {sign} \!\Bigl ( \frac{\nabla _{\textbf{X}^{(i)}_j} f^c\bigl (\textbf{X}^{(i)}_j\bigr )}{\bigl |\nabla _{\textbf{X}^{(i)}} f^c\bigl (\textbf{X}^{(i)}\bigr )\bigr |} \Bigr ) \Bigr ) \quad \text {([6])} \end{aligned}$$

*AttExplore.* AttExplore enhances DNN explainability by integrating model parameter exploration, untargeted adversarial perturbations, and frequency-based input alterations. Starting with a sample image $\textbf{X}^{(i)}$, Gaussian noise $\mathscr {N}(0,1)$ scaled by a perturbation rate $\epsilon /255$—with $\epsilon$ governing the noise magnitude—is added to the spatial domain to inject controlled variability, enabling the exploration of subtle feature sensitivities. The result is transformed into the frequency domain via the Discrete Cosine Transform (DCT), where multiplicative Gaussian noise $\mathscr {N}(1, \sigma )$ modulates the amplitude of each frequency component, controlling spectral patterns relevant to the model’s prediction. The inverse DCT (IDCT) maps the perturbed representation back to the spatial domain, yielding a frequency-aware input $\textbf{X}^{(i)}_{\text {pert}}$ (Eq. 4). AttExplore generates $N$ such variants per input and computes the gradient of the model loss $L(\cdot , y^{(i)})$ with respect to each $\textbf{X}^{(i)}_{\text {pert}}$, where $y^{(i)}$ is the class label. These gradients are aggregated via a nonlinear integration path (Eq. 5), where $\eta$ controls the integration step size (learning rate) and $\odot$ denotes the Hadamard product. This path enables effective exploration of the decision boundary using minimal but informative perturbations, producing stable and focused attributions by avoiding repeated crossings of boundaries associated with non-true classes, which is the main drawback of AGI.

4 $$\begin{aligned}&\textbf{X}^{(i)}_{\text {pert}} = \text {IDCT} \Bigl ( \text {DCT}\bigl (\textbf{X}^{(i)} + \mathscr {N}(0, 1) \cdot \tfrac{\epsilon }{255}\bigr ) * \mathscr {N}(1, \sigma ) \Bigr ) \quad \text {([3])} \end{aligned}$$

5 $$\begin{aligned}&A = \int \Bigl (\eta \cdot \text {sign}\!\Bigl (\frac{1}{N} \sum _{m=1}^N \tfrac{\partial L(\textbf{X}^{(i)}_{\text {pert}}, y^{(i)})}{\partial \textbf{X}^{(i)}_{\text {pert}}}\Bigr )\Bigr ) \odot \Bigl (\frac{1}{N} \sum _{m=1}^N \tfrac{\partial L(\textbf{X}^{(i)}_{\text {pert}}, y^{(i)})}{\partial \textbf{X}^{(i)}_{\text {pert}}}\Bigr ) \, dt \quad \text {([3])} \end{aligned}$$

*Evaluation of the attribution methods.* We evaluate DeepLIFT, AGI, and AttEXplore using both qualitative and quantitative assessments. Qualitatively, heatmaps are visually analysed to determine whether highlighted regions align with clinically meaningful structures, such as neovascularization in OCTA or hyporeflective cystic lesions in OCT B-scans. Quantitatively, the insertion score metric measures how reintroducing pixels, ranked by importance according to each attribution method’s ranking of pixel contributions, affects the model’s predicted probability for the target class (Eq. 6)^41^. Pixels are gradually inserted in order of relevance according to the attribution method. The insertion score measures how quickly the model recovers its original prediction as pixels are added based on their attributed importance. The better the attribution method, the faster the target class probability increases, since the most important regions are inserted first. Consequently, the higher the insertion score indicates the better the attribution method. Let $f(\cdot )$ denote the model’s output probability for the true class $y^{(i)}$ given input image $\textbf{X}^{(i)}$. Then, $f(\textbf{X}^{(i)}_k, y^{(i)})$ is the predicted probability for the true class after revealing the top $k$ most important pixels of $\textbf{X}^{(i)}$. Here, $K$ is the total number of added pixels, and $\Delta k$ is the step size. A higher insertion score suggests that the attributed pixels contribute meaningfully to correct predictions. Complementary to insertion, the deletion score quantifies how the model’s predicted probability changes as pixels are progressively removed in order of decreasing importance (Eq. 7). Starting from the full image, pixels deemed most relevant by the attribution method are successively masked out, yielding intermediate images $\tilde{\textbf{X}}^{(i)}_k$, and the area under the resulting probability–deletion curve is computed. An effective attribution causes a rapid drop in the target-class probability when top-ranked pixels are deleted, so lower deletion scores indicate better attribution performance. Together, high insertion scores and low deletion scores provide a more complete quantitative characterization of attribution quality. This combined evaluation provides a robust assessment of attribution methods in deep learning models.

6 $$\begin{aligned}&\text {Insertion Score} = \sum _{k=1}^{K} f\bigl (\textbf{X}^{(i)}_k, y^{(i)}\bigr ) \cdot \Delta k \end{aligned}$$

7 $$\begin{aligned}&\text {Deletion Score} = \sum _{k=1}^{K} f\bigl (\tilde{\textbf{X}}^{(i)}_k, y^{(i)}\bigr ) \cdot \Delta k \end{aligned}$$

## Results

In this chapter, we present the results of evaluating three attribution methods, DeepLIFT, AGI, and AttEXplore, on two clinically relevant ophthalmic tasks: DR classification using widefield OCTA en face images and retinal fluid detection using OCT B-scans. Each method was applied to the same VGG16-based underlying classifier, and the generated attribution maps were assessed using both quantitative and qualitative measures. We first describe the experimental setup and implementation, followed by a detailed report of classification performance, quantitative attribution evaluation, and visual analysis of heatmaps, concluding with a cross-method comparison.

### Experiments

We conducted a comparative analysis of attribution performance on two distinct medical imaging datasets to assess method robustness across varying visual and pathological patterns. The OCTA dataset captures complex vascular abnormalities associated with DR, such as neovascularization and non-perfusion, in high-resolution en face views. In contrast, the OCT B-scan dataset focuses on fluid accumulation patterns that might indicate macular edema. These datasets differ in anatomical focus and image structure, planar OCTA projections versus cross-sectional OCT B-scans, providing a complementary testbed to assess attribution generalization across modalities. We applied each method within a shared VGG16 classifier and evaluated attribution quality through the insertion and deletion score metrics and visual heatmap analysis, allowing for a comprehensive examination of clinical relevance and model trustworthiness.

**Implementation details.** Our implementation framework was developed using Python 3.11 and TensorFlow 2.18. All training and inference tasks were performed on an NVIDIA GeForce RTX 4090 GPU with 24 GB of memory. Hyperparameters for all models were set, without extensive optimization. The reported scores are from the models exhibiting the highest performance on the test dataset. The list of hyperparameters to train our models used for all experiments includes a learning rate of 1e-5, a batch size of 1, the relu activation function, the adam optimizer, 256 neurons in the final dense layer, a dropout rate of 0.5, and the binary focal cross-entropy loss function. Moreover, for DeepLIFT, we used a noisy background as described by^3^. For AGI, we set the learning rate ($\eta$) to 0.05 and the maximum number of steps to 20, following the approach in^6^. For AttEXplore, we configured the number of approximate features (N) to 20, the standard deviation of Gaussian noise ($\sigma$) to 16, the perturbation rate ($\epsilon$) to 48/255, and the maximum number of steps to 10, as suggested in^3^. Additionally, we performed extensive hyperparameter tuning for the learning rate and epsilon, determining optimal values of 0.05 and 48/255, respectively. The effects and sensitivity of these parameters are described in more detail in the Supplementary Material (Hyperparameter Sensitivity). All heatmaps presented in the results section were generated using the specified hyperparameters.

**Binary classification model performance.** Before performing attribution analysis, we first evaluated the VGG16 backbone on each task to ensure reliable predictions. Performance was assessed using standard classification metrics, accuracy, sensitivity, specificity, precision, F1 score, area under the ROC curve (AUC), and average precision, computed on OCTA en face and B-scan datasets. For DR classification using widefield OCTA en face images, we implemented a 5-fold cross-validation strategy. To prevent data leakage, all scans from a given patient were assigned to a single data partition (training, validation, or testing) throughout the evaluation.

**Quantitative evaluation of attribution maps.** We first apply each attribution method to the VGG16 classifier’s outputs on both the OCTA en face and OCT B-scan datasets, then use the insertion score (Eq. 6) to measure how rapidly model confidence recovers as we reintroduce the most salient pixels. By running this evaluation on k (in our experiments, k=50) randomly selected images per dataset based on the insertion score, we can directly reveal how well each method identifies truly important regions in two imaging modalities. This approach not only pinpoints which attribution methods produce steeper insertion curves overall, but also highlights their differential sensitivity to the planar vascular morphology of OCTA en face images versus the layered, cross-sectional fluid features in OCT B-scans. We also repeat the same experiment on 50 randomly selected ImageNet samples as a sanity check to confirm the correctness and stability of our attribution method implementations. In parallel, we compute the deletion score (Eq. 7), which quantifies how rapidly the target-class probability drops as the most relevant pixels are progressively removed. Lower deletion scores indicate that top-ranked regions are truly critical to the prediction, providing a counterpoint to insertion’s recovery behavior. Considered together, high insertion and low deletion jointly signal faithful attributions across both OCTA en face and OCT B-scan modalities.

**Qualitative evaluation of attribution maps.** We qualitatively evaluate the attribution methods through visual analysis, complementing the earlier quantitative assessment. This allows us to examine whether the insertion and deletion scores align with the visual effectiveness of the heatmaps in identifying clinically relevant regions. We primarily use the insertion scores to interrogate the visual assessment, as insertion is considered the more decisive quantitative metric^3,6^, with deletion scores included as a complementary quantitative reference. We overlay each method’s attribution map onto the original OCTA en face image for DR classification and the OCT B-scan for fluid detection. These heatmaps are generated for a stratified random subset of cases, including both DR-positive and DR-negative subjects in the OCTA dataset, and B-scans with and without retinal fluid in the OCT dataset. Visual heatmap analysis is then used to assess the spatial alignment between high-attribution areas and known pathological or anatomical structures (e.g., neovascularization in OCTA and fluid pockets in B-scans). This process ensures an unbiased, clinical validation of each method’s ability.

### Results

We first present VGG16 classification results for both DR classification and fluid detection. We also present results from two complementary analyses of attribution performance. The first is a quantitative analysis using insertion and deletion scores to assess how rapidly each method’s top-ranked pixels influence model confidence in OCTA en face and OCT B-scan images. The second is a qualitative assessment in which visual heatmap analysis is used to verify alignment with known pathologies. Finally, we outline a cross-method comparison that previews each technique’s strengths and limitations across both imaging modalities.

**Quantitative results.** Here, we detail classifier performance across both datasets and evaluate attribution methods using the insertion and deletion scores.

*Widefield OCTA en face images.* Across five-fold cross-validation, VGG16 achieved a mean accuracy of 0.940, with a range of 0.928 to 0.942 across the folds. The model yielded a sensitivity of 0.950 (0.902–0.980), specificity of 0.911 (0.833–1.000), precision of 0.969 (0.943–1.000), F1-score of 0.959 (0.948–0.961), AUC of 0.930 (0.907–0.951), and average precision of 0.956 (0.940–0.974), where the values in parentheses indicate the minimum and maximum scores observed across folds.

*Kermany OCT B-scans.* For fluid detection on the preprocessed Kermany OCT B-scan dataset, VGG16 achieved an accuracy of 0.980, sensitivity of 0.950, specificity of 0.996, precision of 0.993, F1-score of 0.971, AUC of 0.973, and average precision of 0.962 on the samples of the test set. These evaluations show that the classifier provides robust discrimination of DR versus non-DR and fluid versus non-fluid cases, forming a solid foundation for subsequent attribution analysis.

*Insertion and deletion score results.* Table 1 reports the insertion and deletion scores computed using VGG16 across the datasets, Kermany and widefield OCTA, for three attribution methods: DeepLIFT, AGI, and AttExplore. ImageNet is also included as a baseline for performing a sanity check. Scores were obtained from a randomly selected subset of 50 samples per dataset. For ImageNet, DeepLIFT yielded 0.088 (range: 0.001–0.78), AGI 0.265 (0.003–0.966), and AttExplore 0.312 (0.004–0.984), closely aligning with the original AttExplore paper, which reported 0.082, 0.258, and 0.318, respectively. This confirms the correctness of our implementation. For Kermany, DeepLIFT achieved 0.828 (0.707–0.924), AGI 0.877 (0.619–0.968), and AttExplore 0.868 (0.545–0.977). For widefield OCTA, DeepLIFT scored 0.787 (0.413–0.996), AGI 0.901 (0.544–0.991), and AttExplore 0.938 (0.664–0.997). These values indicate consistent performance trends across datasets, with AttExplore generally achieving the highest scores, AGI performing competitively, and DeepLIFT yielding lower values. Complementing the insertion results, the deletion scores provide an additional perspective on attribution quality. For ImageNet, all methods yield low deletion scores, with DeepLIFT achieving the lowest value at 0.023 (range: 0–0.344), followed by AttExplore at 0.034 (0–0.436) and AGI at 0.038 (0–0.485). On the Kermany dataset, AGI obtains the lowest deletion score at 0.600 (0.377–0.682), compared to AttExplore at 0.640 (0.353–0.718) and DeepLIFT at 0.740 (0.449–0.923). Similarly, for widefield OCTA, AGI again reports the lowest deletion score at 0.658 (0.580–0.719), relative to AttExplore at 0.680 (0.554–0.749) and DeepLIFT at 0.790 (0.443–0.926).

**Table 1:** Insertion and deletion scores (mean [range]) for three attribution methods—DeepLIFT (Deep Learning Important FeaTures), AGI (Adversarial Gradient Integration), and AttEXplore (Attribution method for Explanation with model parameter eXploration)—evaluated on VGG16 across ImageNet (implementation sanity check), Kermany OCT B-scans, and widefield OCTA en face images.

Method/Dataset	ImageNet		Kermany		Widefield OCTA	
	Insertion score	Deletion score	Insertion score	Deletion score	Insertion score	Deletion score
DeepLIFT	0.088 (0.001, 0.78)	0.023 (0, 0.344)	0.828 (0.707, 0.924)	0.740 (0.449, 0.923)	0.787 (0.413, 0.996)	0.790 (0.443, 0.926)
AGI	0.265 (0.003, 0.966)	0.038 (0, 0.485)	0.877 (0.619, 0.968)	0.600 (0.377, 0.682)	0.901 (0.544, 0.991)	0.658 (0.580, 0.719)
AttEXplore	0.312 (0.004, 0.984)	0.034 (0, 0.436)	0.868 (0.545, 0.977)	0.640 (0.353, 0.718)	0.938 (0.664, 0.997)	0.680 (0.554, 0.749)

**Qualitative results.** We present qualitative results for the widefield OCTA and Kermany OCT datasets separately.

**Fig. 3:**
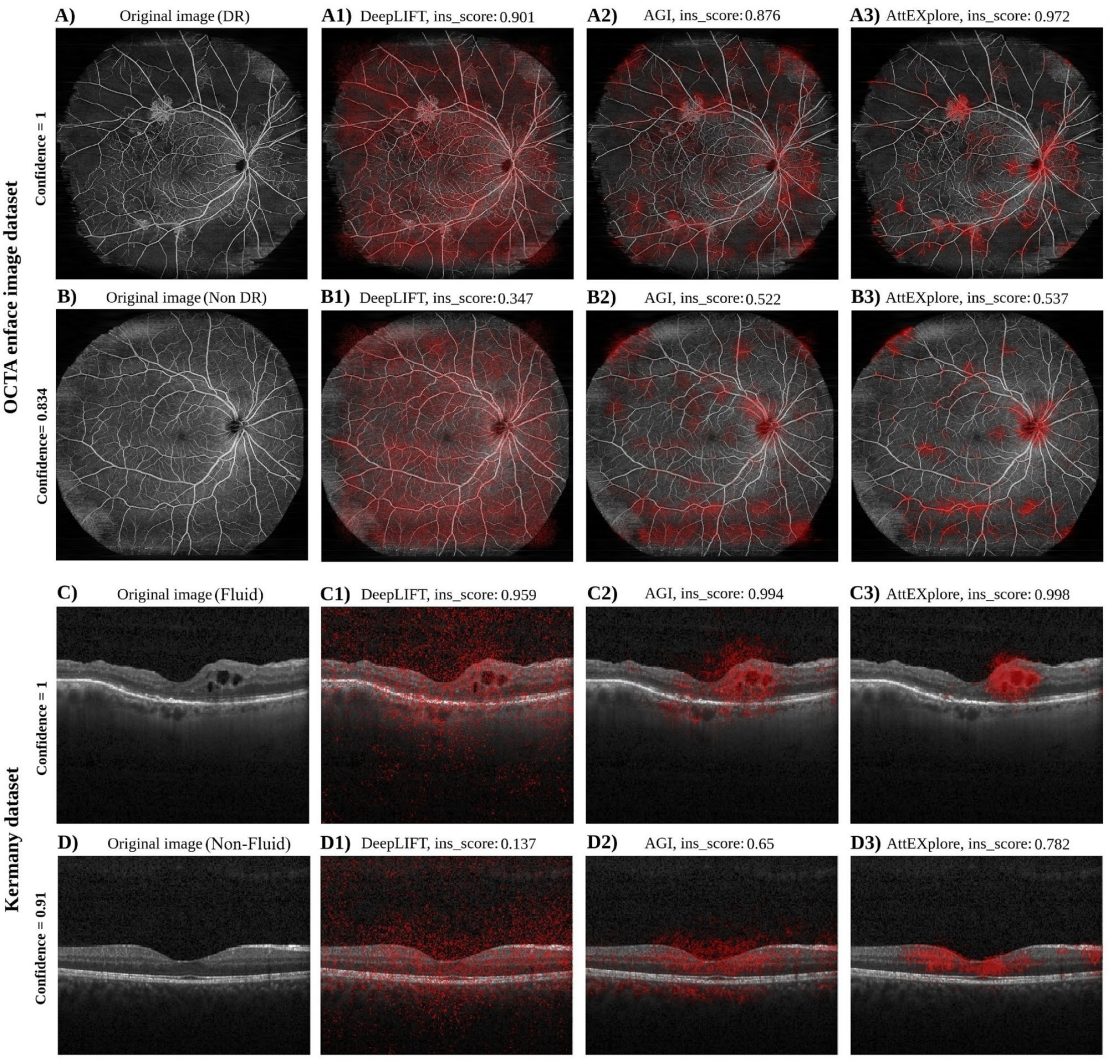
Comparison of attribution methods on OCTA en face images and OCT B-scans, with (**A**, **B**) showing images from a DR patient and a non-DR subject, and (**C**,**D**) representing a fluid and a non-fluid B-scan, respectively. Each method—DeepLIFT (Deep Learning Important FeaTures), AGI (Adversarial Gradient Integration), and AttEXplore (Attribution method for Explanation with model parameter eXploration)—exhibits different focus in capturing relevant image regions, with differences in the specificity and diagnostic relevance of the resulting attribution maps across pathological and non-pathological cases. Ins_score = insertion score (area under model confidence vs. fraction revealed).

*Widefield OCTA en face images.* Fig. 3 (A) and (B) show OCTA en face images of a DR case and a non-DR subject, respectively, which are analyzed using the attribution methods. For the DR case, DeepLIFT (A1) produces non-specific attributions that make it difficult to pinpoint key pathological regions, whereas AGI (A2) provides a structured heatmap covering NV regions and additionally highlights areas near the optic nerve. AttExplore (A3) generates more focused and structured attributions on NV regions than AGI and also covers some IRMA areas, while similarly highlighting the optic nerve region. For the non-DR subject in Fig. 3B, the image shows well-formed retinal vasculature. Because no irregular patterns are present, all methods would be expected to produce evenly distributed attribution values across the retina, reflecting a potential interpretation of attributions in the context of normal anatomical features. DeepLIFT, in particular, shows the least heterogeneous – and thus the best – distribution compared to AGI and AttExplore. However, the problem here is that this distribution is almost identical to that in the DR case. AGI and AttEXplore highlight several vascular regions, although their activations remain somewhat dispersed, particularly toward the periphery. It is also worth mentioning that artifacts within OCTA volumes can generate false biomarkers, leading attribution methods to highlight areas that do not reflect true lesions. In Fig. 3A, we observe attention in the superotemporal peripapillary region, particularly pronounced with AGI. Additionally, all methods exhibit a similar attention pattern toward artifacts in the inferotemporal peripapillary region, as shown in Fig. 3B. Moreover, the horizontal stripe-like activations along the inferior retina in Fig. 3B, especially with AGI and AttEXplore, might suggest a consistent model bias or systematic attribution pattern that reflects sensitivity to imaging artifacts rather than true pathology.

*Kermany OCT B-scans.* Fig. 3 (C) and (D) display two OCT B-scan images evaluated using the attribution methods: (C) depicts a patient with retinal fluid (visible as sharply defined dark areas within the retina), and (D) shows a non-fluid patient. For the fluid B-scan in Fig. 3-C, DeepLIFT $(\text {C1})$ generates scattered attributions across the retina, vitreous, and choroid, providing limited localization of the fluid pathology. AGI $(\text {C2})$ better pinpoints the general area of pathology, though some dispersion around the choroid and vitreous persists. AttEXplore $(\text {C3})$ provides the most precise attribution in terms of clinical relevance, highlighting the fluid regions. In the non-fluid OCT B-scan (Fig. 3-D), DeepLIFT $(\text {D1})$ yields diffuse attributions across the image, very similar to those in the fluid case. AGI $(\text {D2})$ highlights a more centralized area but retains some dispersion, and AttEXplore $(\text {D3})$ demonstrates a sharper focus around the foveal dip. This means that the model might learn a pronounced foveal-dip bias in non-fluid cases, attending almost exclusively to the area surrounding the foveal depression as its primary indicator of healthy, fluid-free retina. In addition, it is worth noting that AGI—and to an even greater extent, DeepLIFT—produce attributions outside the retina, i.e., in non-informative background regions.

**Fig. 4:**
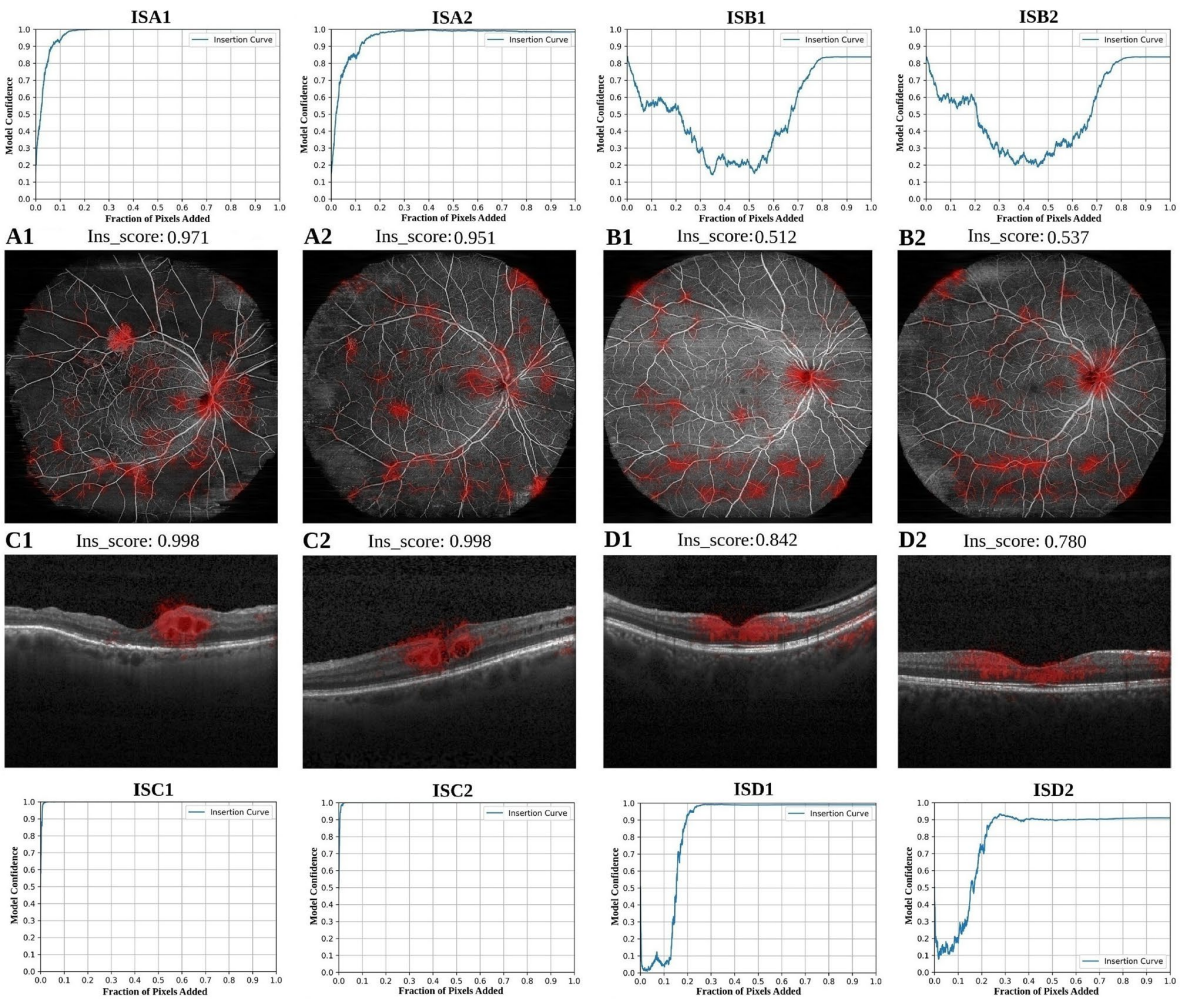
Insertion score (IS) plots and attribution map overlays for OCTA en face images (top: ISA1, ISA2 – DR; ISB1, ISB2 – non-DR) and OCT B-scans (bottom: ISC1, ISC2 – fluid B-scans; ISD1, ISD2 – non-fluid B-scans), demonstrating differences in model confidence and attribution patterns as evaluated by AttEXplore (Attribution method for Explanation with model parameter eXploration). Ins_score = insertion score (area under model confidence vs. fraction revealed).

**Relating visual explanations and their corresponding insertion scores.** We explore the relationship between visual attribution maps and their corresponding insertion scores to assess how well the insertion score aligns with clinically relevant regions. While the heatmaps in Fig. 3-A, even for the best-performing method, AttEXplore, omit ischemic (or NPA) regions, their insertion scores remain high (0.972). These high values suggest that the underlying attribution methods can identify the most relevant image regions that contribute most to a correct and stable classification. However, this does not necessarily imply that the resulting heatmaps are clinically meaningful, as these features may reflect latent patterns rather than clinically interpretable structures. Moreover, DeepLIFT shows a higher insertion score than AGI, even though its visualizations are even less helpful than those of AGI. Similarly, for the fluid B-scan in Fig. 3-C, the high insertion scores for AGI (0.994) and AttEXplore (0.998) indicate attributions that support the model’s correct classification and, in these cases, overlap with clinically relevant fluid regions. In contrast, DeepLIFT’s comparably high score (0.959) occurs despite producing less clinically meaningful attribution. Taken together, these examples underscore that a high insertion score alone is insufficient for assessing clinical interpretability and should be complemented by visual explanations (e.g., heatmaps). In pathological cases, the task might resemble an object-detection problem (especially in the Kermany dataset), similar to that in natural image settings, where the presence of localized abnormalities (e.g., DR signs or fluid pockets) allows the model to base its prediction on relatively small, salient regions. This can make the heatmaps and insertion score more informative. Conversely, for the non-pathological images in Fig. 3 (B and D), we observe lower insertion score values. In normal cases, however, the classifier must effectively inspect the full image to confirm the absence of abnormalities, since a single DR sign or fluid pocket could flip the prediction to abnormal. As a result, the model’s confidence tends to fluctuate or remain low until global contextual pixels are inserted to reveal a sufficiently complete vascular or structural pattern. This reveals the importance of differentiating between pathological and non-pathological cases when interpreting insertion scores. This is because the metric behaves fundamentally differently depending on whether the prediction relies on localized abnormalities or on the absence of such findings across the entire image.

Fig. 4 presents insertion score plots and corresponding attribution map overlays for both OCTA en face images (top rows: A1–B2, ISA1–ISB2) and OCT B-scans (bottom rows: C1–D2, ISC1–ISD2), analyzed using AttEXplore. The figure investigates differences in insertion score curves and attribution maps between pathological and non-pathological images in OCTA and OCT. In the pathological cases, DR (A1, A2; ISA1, ISA2) and fluid (C1, C2; ISC1, ISC2), the insertion curves rise steeply and stabilize early with high scores (all above 0.95), indicating that the attributed pixels (regions) are sufficient to support a correct and stable classification by the model. In contrast, the non-pathological images, non-DR (B1, B2; ISB1, ISB2) and non-fluid (D1, D2; ISD1, ISD2), exhibit different insertion curve behaviors. For non-DR, the model’s confidence is initially high but decreases as more pixels are added, rising again only after a majority of the image is revealed (almost 70% of pixels). For non-fluid cases, confidence starts at high levels, is immediately followed by a steep drop, and only increases after inserting almost 10% of pixels, plateauing thereafter. These patterns are consistent with the notion that, for normal cases, the classifier must effectively inspect the full image to confirm the absence of pathology. Consequently, insertion scores for non-pathological images tend to be lower and less smooth.

**Fig. 5:**
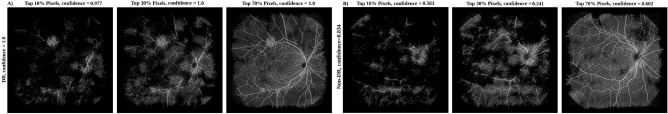
Intermediate reconstruction steps during insertion for a DR case (**A**) and a non-DR case (**B**), showing model confidence at 10%, 30%, and 70% of pixels inserted based on attribution ranking.

Fig. 5 illustrates how intermediate stages of pixel insertion (10%, 30%, 70%) affect model confidence and attribution in DR and non-DR OCTA en face image recomposition. Each panel displays the image progression alongside the corresponding model confidence scores at these stages. For the DR case (A), after inserting only 30% of pixels, the model’s confidence reaches its maximum (1.0), reflecting early identification of irregular vascular patterns like the neovascularization toward the center-left, which is even visible when 10% of pixels are inserted. Conversely, in the non-DR case (B), the model initially predicts the correct class with moderate confidence (0.583 at 10%). However, as more pixels are added, the confidence drops to 0.241 at 30%. This occurs because sparse insertion from a black baseline creates discontinuous vasculature and large dark regions that can visually resemble pathological patterns, making it difficult for the model (and even clinicians) to recognise normal anatomy until sufficient global context is present. Only after inserting approximately 70% of pixels, the model’s confidence correctly rises to 0.602, indicating that the model begins to recognize the vascular pattern as sufficiently complete. This underscores the value of inspecting intermediate insertion steps, as they provide intuition about how the insertion score develops. This is particularly important in normal cases, where confirmation of normality depends on integrating information across the entire image.

## Discussion

In this study, we evaluated three state-of-the-art attribution methods using a VGG16-based model in two ophthalmic image analysis tasks: (1) DR classification utilizing a widefield OCTA en face image dataset and (2) fluid detection based on an OCT B-scan dataset. The comparative analysis includes combining quantitative assessment via insertion scores with qualitative evaluation through visual analysis of pixel-level heatmaps to examine each method’s ability to highlight clinically relevant regions. Despite the effectiveness of methods like AttEXplore and AGI in generating meaningful attributions for natural images, their performance in the medical imaging domain remains unreliable. Even after extensive tuning, they fail to produce consistently reliable results across and within the studied imaging modalities. This might be due to the following reasons:

**Limitations of attribution maps as pixel-accurate segmentations.** Expecting exact heatmaps from attribution methods is unrealistic, as these methods are fundamentally designed to reflect the model’s decision process rather than precise localization. This indicates that their resulting attribution maps emphasize influential regions without guaranteeing pixel-level accuracy. As highlighted in^42^, attribution methods primarily provide insight into model behavior rather than producing segmentations that perfectly align with human annotations. This makes direct comparisons with segmentation masks inherently flawed. In clinical applications, this means that attribution heatmaps should not be used as substitutes for expert-annotated segmentations, as they may highlight regions irrelevant to diagnosis or overlook clinically significant findings. Therefore, their use for guiding diagnosis or treatment decisions should be approached with caution and always supplemented by clinical validation.

**Method-dependent attribution variability in a fixed network.** Although all three attribution methods, DeepLIFT, AGI, and AttEXplore, are applied to the same underlying trained VGG16, they produce distinct heatmaps due to their fundamentally different mechanisms for assigning feature importance. DeepLIFT relies on reference-based activation differences, AGI traverses adversarial paths to explore decision boundaries, and AttEXplore incorporates the frequency-based adversarial attacks to find the attributions. These methodological differences lead each approach to highlight different aspects of the input image, resulting in variability in the generated attribution maps despite the shared model architecture. This is problematic in clinical settings, since different attribution methods can highlight disparate regions for the same prediction. This undermines trust and consistency, and indicates that current attribution methods may not yet be sufficiently robust or reliable for use in routine clinical decision support.

**Attribution methods do not rank image regions like clinicians.** Attribution methods do not necessarily rank image regions in the same way expert clinicians do, as their highlighted areas stem from the internal representations learned by the network rather than explicit clinical knowledge. These regions often emerge from the latent feature space of the neural network, capturing abstract patterns that may not directly correspond to anatomical or pathological landmarks. Consequently, the resulting heatmaps can reflect model decision-relevant patterns that diverge significantly from a clinician’s visual reasoning or diagnostic focus. This finding implies that if attribution methods are used for interpreting clinical decision support algorithms, there is a risk that highlighted regions may not correspond to clinically meaningful features. This could potentially lead to misinterpretation or misplaced trust in algorithmic decisions. Thus, careful validation and clinician oversight are essential before integrating these methods into clinical workflows.

**Quantitative metrics do not reflect clinical relevance.** The insertion score alone does not guarantee meaningful clinical explanations, as it measures the increase in model confidence when highlighted pixels (or regions) are revealed. However, it does not assess whether those pixels (or regions) are visually or clinically relevant, nor does it measure clinical interpretability. In pathological cases, such as DR and fluid examples, we observe insertion curves that rise steeply and stabilise early with high scores (all above 0.95). These patterns suggest that a relatively small set of attributed pixels can support a correct and stable classification. In such settings, the task can resemble an object-detection problem, where localized abnormalities (e.g., DR signs or fluid pockets) drive the prediction. This can make both the heatmaps and insertion scores informative for understanding how the model uses these regions. However, the omission of ischemic (or NPA) areas in some DR cases despite high insertion scores shows that such behaviour does not necessarily imply that the resulting heatmaps are clinically complete or fully meaningful. Conversely, in non-pathological images, insertion scores tend to be lower and the corresponding curves less smooth. This behaviour is consistent with the nature of the task: to confirm normality, the classifier effectively needs to inspect the full image, since a single DR sign or fluid pocket could flip the prediction to abnormal. When pixels are inserted from a black baseline, intermediate reconstructions can temporarily resemble pathological patterns. This leads to fluctuations in confidence until sufficient global context is available. As a result, insertion scores for normal cases behave differently, and the corresponding heatmaps may be less clinically informative. This highlights the importance of differentiating between pathological and non-pathological cases when interpreting insertion scores. From a purely quantitative perspective, deletion scores broadly follow the same pattern as insertion scores. AGI achieves the lowest (i.e., most favourable) deletion values on the Kermany and widefield OCTA datasets. This suggests that AGI and AttEXplore generally align more closely with the model’s internal notion of importance than DeepLIFT on these datasets. However, insertion and deletion scores mainly reflect how attributions affect model confidence, rather than providing a direct measure of correspondence with pathological structures. Strong quantitative performance, therefore, does not necessarily translate into clinically meaningful or trustworthy explanations. Overall, insertion and deletion scores are best viewed as complementary indicators of model behaviour that should be interpreted alongside qualitative visual assessments, clinical context, and the distinction between pathological and non-pathological cases.

**Hyperparameter sensitivity.** DeepLIFT’s effectiveness heavily depends on the selection of a reference input, which serves as a baseline for attribution. This baseline does not always represent a truly neutral state, and an inappropriate choice can lead to misleading or less interpretable attributions. While DeepLIFT provides insights into feature importance, it is a relatively traditional method that relies on backpropagation and is highly sensitive to baseline selection. This sensitivity limits its robustness, particularly in complex medical imaging tasks such as OCT and OCTA analysis, where other advanced attribution techniques may offer greater precision and clinical relevance^5,6^. Methods such as AGI and AttEXplore provide pixel-level explanations to enhance model transparency; however, their effectiveness is highly dependent on hyperparameter selection. In AGI, parameters like the learning rate ($\eta$) and the maximum number of steps directly affect how well the method traverses decision boundaries and stabilizes attribution computations. Similarly, AttEXplore’s performance is sensitive to the choice of $\eta$, $\epsilon$ (perturbation rate), $\sigma$ (frequency noise strength), and *N* (number of approximate features), which collectively determine the trade-off between capturing fine-grained details and avoiding excessive noise in the attribution maps. The high sensitivity of DeepLIFT, AGI, and AttEXplore to baseline inputs and hyperparameter settings poses significant challenges in clinical applications by undermining the trustworthiness of their attributions.

In general, while attribution methods like AttEXplore, AGI, and DeepLIFT provide insight into model behavior, their outputs vary significantly due to differences in underlying assumptions, reliance on latent representations, and sensitivity to hyperparameters. Importantly, these methods do not localize features in the same way clinicians do, as their highlighted regions often reflect abstract patterns from the model’s latent space rather than anatomical or pathological landmarks. Moreover, it holds limited value to fine-tune methods designed primarily to explain the underlying model rather than to be optimized themselves. Despite robust classification performance, our studied attribution methods for the DR vs. non-DR task did not yield clinically convincing explanations. This suggests that, even with tuning, explainability methods may fall short, highlighting the need for balanced qualitative and quantitative evaluation in ophthalmic applications. In addition, AttEXplore has 5 main hyperparameters compared to AGI’s 3, significantly impacting the resulting heatmaps. Thus, reducing the number of hyperparameters or automatically tuning them would be beneficial for clinical applications, as simpler or self-tuned methods improve reproducibility, robustness, and ease of integration into clinical workflows. Taken together, these observations raise the practical question of which method should be preferred in real-world use. In considering which attribution method should be preferred in practical use, our findings show that although AGI and AttEXplore achieve similarly strong quantitative performance, AttEXplore consistently provides clearer and more clinically aligned visual explanations for diseased (lesion) class than AGI, especially in the Kermany dataset. DeepLIFT, by contrast, performs less favorably in both quantitative and qualitative assessments. Notably, even for the best-performing methods, quantitative metrics such as insertion and deletion scores mainly describe how attributions influence model confidence and do not directly reflect clinical relevance. Consequently, attribution methods should be selected and interpreted primarily based on their qualitative alignment with known pathology, taking into account clinical context and the distinction between pathological and non-pathological cases. Furthermore, employing foundation models pre-trained with self-supervised learning may broaden the applicability of attribution methods, as these models provide stable and semantically rich feature representations. While our primary evaluation focuses on VGG16, the same procedures can be applied directly to modern vision foundation models without architectural modification. To illustrate this generalizability, we additionally applied AttExplore to the encoder of the RETFound vision foundation model. Qualitative examples are included in the Supplementary Material (Applicability of attribution methods on vision foundation models).

## Supplementary Information

Supplementary Information.

## Data Availability

The OCTA dataset used for the study originates from a clinical study and cannot be made publicly available due to patient privacy considerations and GDPR regulations, as the retinal images contain biometric information. The OCT B-scan dataset is publicly accessible and can be obtained from the following source: https://www.kaggle.com/datasets/paultimothymooney/kermany2018.
