# Supplementary material for: Applicability study of AI attribution methods for ophthalmic image classification
Source: Sci Rep. 2026 Jan 5;16:3296. doi: 10.1038/s41598-025-33120-5 (PMC12835177; doi:10.1038/s41598-025-33120-5)
Supplement: Supplementary file 1 — Supplementary Information. [file 41598_2025_33120_MOESM1_ESM.pdf]

Supplementary Material for

Applicability study of AI attribution methods for ophthalmic image classification

Ali Yavari[1,*], Tilman Schmoll[1,2], Rainer A. Leitgeb[1], Kim Lien Randall Huber[3], Heiko Stino[3], Andreas Pollreisz[3], Wolfgang Drexler[1], and Thomas Schlegl[1]

[1]Center for Medical Physics and Biomedical Engineering, Medical University of Vienna, Waehringer Guertel 18-20 (4L), 1090, Vienna, Austria
[2]Carl Zeiss Meditec AG, Oberkochen, Germany
[3]Department of Ophthalmology and Optometry, Medical University of Vienna, Waehringer Guertel 18-20, 1090 Vienna, Austria
[*]ali.yavari@meduniwien.ac.at

Hyperparameter Sensitivity

Fig. 1 illustrates how variations in the hyperparameters ε and η influence attribution quality and lesion localization in AttEXplore. The attribution maps, generated for both an OCTA en face image (A-DR) and an OCT B-scan (B-fluid), demonstrate the need for cautious hyperparameter tuning, as improper settings may amplify irrelevant patterns rather than potential aligning with clinically meaningful structures. In the OCTA en face image (Fig. 1-A), at low perturbation strength ($\varepsilon = 8/255$), attributions remain tightly localized to small capillary branches. As ε increases ($\varepsilon = 48/255$ and $\varepsilon = 72/255$), the highlighted regions spread over larger vascular networks, including regions of neovascularization. While this broader coverage may offer insight into general vascular structure, it does not necessarily correspond to disease-specific patterns, potentially introducing irrelevant activations. Notably, when $\varepsilon = 48/255$, the attributions distinctly capture two brightened neurovascular regions, suggesting that this setting may enhance sensitivity to abnormal vasculature while maintaining some level of specificity. On the other hand, we observe no substantial changes with varying η, suggesting that this modality may be less sensitive to this parameter. Morerver, the insertion scores provide additional insight into the ε behavior. At the lowest ε (8/255), insertion scores are significantly lower compared to higher values, but they increase as ε rises, reflecting stronger alignment between highlighted regions and model confidence.

For the OCT B-scan (Fig. 1-B), at lower ε values ($\varepsilon = 8/255$), the attributions remain relatively dispersed, with activations extending beyond the core lesion. As ε increases ($\varepsilon = 48/255$ and $\varepsilon = 72/255$), the attributions become more concentrated around the fluid region, indicating sharper localization and improved alignment with clinically relevant features. The effect of η follows a similar trend across lower values (B1: $\eta = 0.0005$, B2: $\eta = 0.005$, B3: $\eta = 0.05$), where attributions remain concentrated within the fluid pockets, maintaining sensitivity in identifying disease-related features. However, at the highest η value (B4: $\eta = 1$), a distinct shift occurs, leading to potentially capturing broader contextual features, particularly around the periphery. This shift might emphasize non-pathological regions, reducing the precision of the method, though the insertion scores for all states remain above 0.975.

Applicability of attribution methods on vision foundation models

To exemplarily evaluate the applicability of attribution methods to more advanced state-of-the-art models, we conducted an additional experiment using the RETFound vision foundation model, a self-supervised ViT-Large model pretrained on 1.6M retinal images [1]. We fine-tuned only the last four encoder blocks while keeping the remaining weights frozen, and trained the model on the curated Kermany OCT B-scan dataset described above for the binary task of fluid versus non-fluid detection. After fine-tuning, attribution maps were computed directly on the model's prediction scores without requiring any modification to the architecture or attribution procedures, demonstrating the plug-and-play compatibility of the methods with third-party pretrained models. This confirms that the attribution pipeline generalizes seamlessly from VGG-based architectures to advanced foundation-model-based feature extractors. Fig. 2 shows attribution maps obtained with AttEXplore applied to the fine-tuned RETFound model on representative fluid and non-fluid B-scans from the Kermany dataset. In non-fluid-scans (right), unlike the pronounced foveal-dip focus observed in the VGG16 results, the attributions do not concentrate around the foveal depression but instead appear more diffusely distributed across the retinal layers. In fluid B-scans (left), the heatmaps just roughly localize the location of single fluid pockets. As discussed in the main text, each

attribution method has its own hyperparameters, and these hyperparameters must also be tuned for the specific underlying classification model. Because the goal of this experiment was to demonstrate the ease of applying attribution methods to more complex architectures, we simply reused the same hyperparameters employed for the VGG16 classifier.

References

1. Zhou, Y., Chia, M. A., Wagner, S. K. *et al.* A foundation model for generalizable disease detection from retinal images. *Nat.* **622**, 156–163, DOI: 10.1038/s41586-023-06555-x (2023).

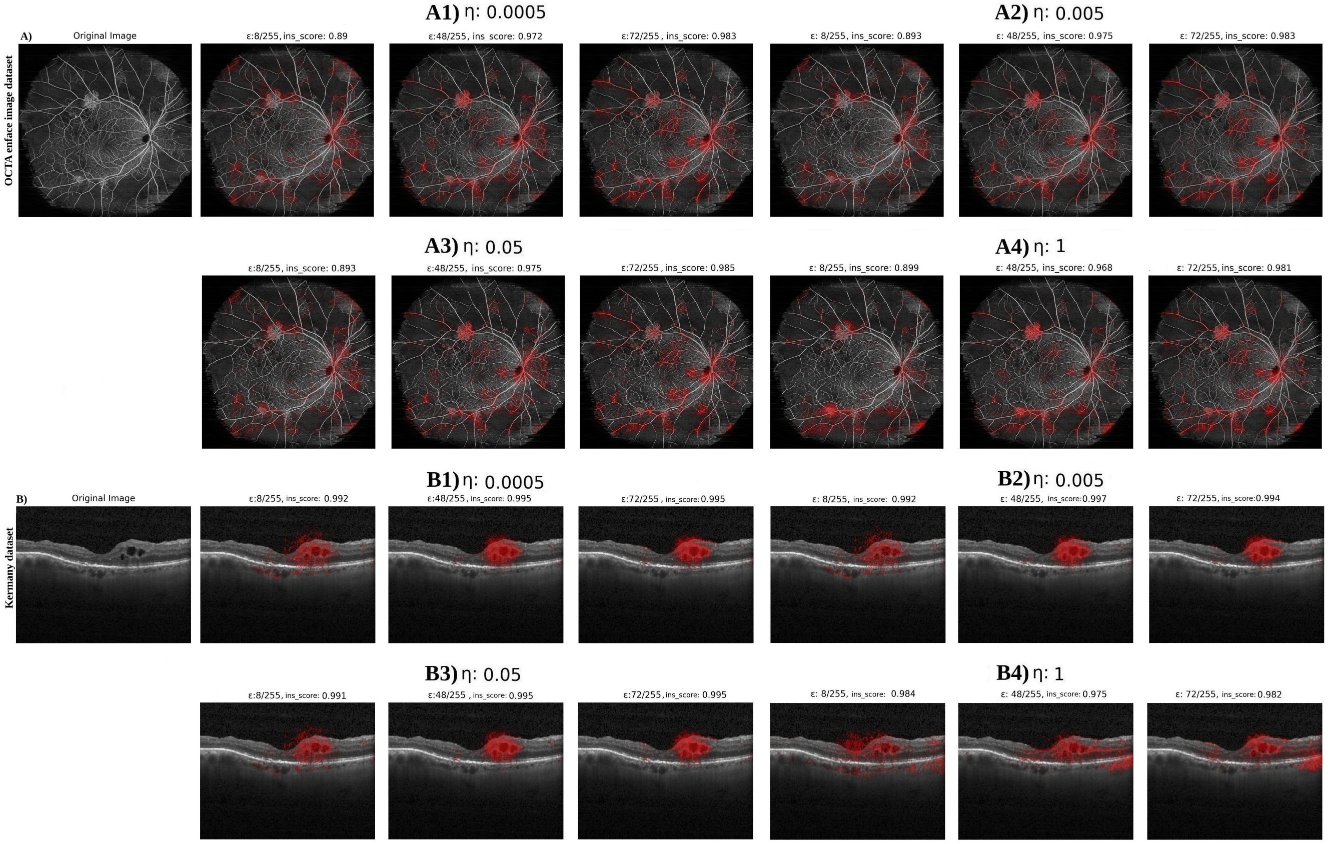

Supplementary Figure 1. Attribution maps generated by AttEXplore for an OCTA en face image (A) and an OCT B-scan (B) under varying perturbation magnitudes (ε) and learning rates (η). For each row (A1–A4, B1–B4), the learning rate η is fixed while the perturbation rate ε increases from left to right: (a) $\varepsilon = 8/255$, (b) $\varepsilon = 48/255$, and (c) $\varepsilon = 72/255$. Rows correspond to different learning rates: A1/B1: $\eta = 0.0005$, A2/B2: $\eta = 0.005$, A3/B3: $\eta = 0.05$, and A4/B4: $\eta = 1$.

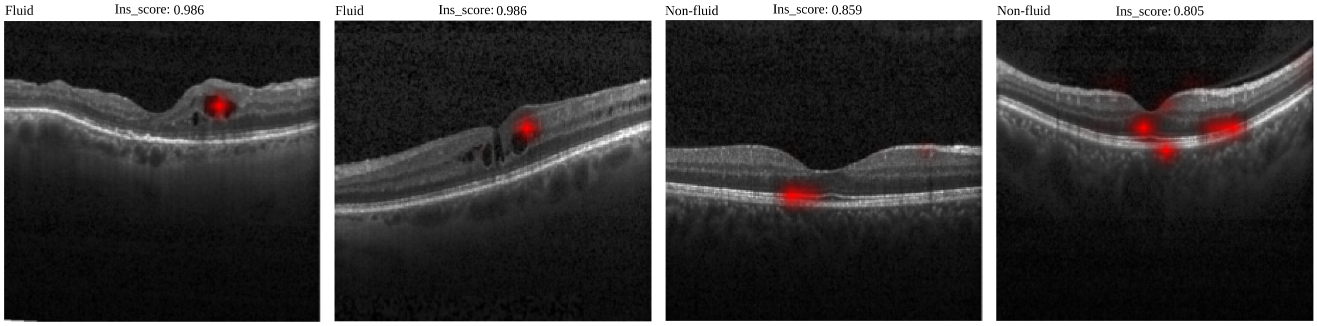

Supplementary Figure 2. Attribution maps generated by AttEXplore ($\varepsilon = 48/255$ and $\eta = 0.05$) applied to the fine-tuned RETFound model on fluid (left two) and non-fluid (right two) B-scans from the Kermany dataset.
